# The dual role of the neuroinflammatory response after ischemic stroke: modulatory effects of hypothermia

**DOI:** 10.1186/1742-2094-7-74

**Published:** 2010-11-01

**Authors:** An-Gaëlle Ceulemans, Tine Zgavc, Ron Kooijman, Said Hachimi-Idrissi, Sophie Sarre, Yvette Michotte

**Affiliations:** 1 Department of Pharmaceutical Chemistry and Drug Analysis, Research Group Experimental Neuropharmacology, Vrije Universiteit Brussel, Laarbeeklaan 103, 1090 Brussels, Belgium; 2 Department of Pharmacology, Vrije Universiteit Brussel, Laarbeeklaan 103, 1090 Brussels, Belgium; 3 Critical Care Department and Cerebral Resuscitation Research Group, Vrije Universiteit Brussel, Brussels, Belgium

## Abstract

Neuroinflammation is a key element in the ischemic cascade after cerebral ischemia that results in cell damage and death in the subacute phase. However, anti-inflammatory drugs do not improve outcome in clinical settings suggesting that the neuroinflammatory response after an ischemic stroke is not entirely detrimental. This review describes the different key players in neuroinflammation and their possible detrimental and protective effects in stroke. Because of its inhibitory influence on several pathways of the ischemic cascade, hypothermia has been introduced as a promising neuroprotective strategy. This review also discusses the influence of hypothermia on the neuroinflammatory response. We conclude that hypothermia exerts both stimulating and inhibiting effects on different aspects of neuroinflammation and hypothesize that these effects are key to neuroprotection.

## Introduction

Inflammation is an essential tool to defend oneself against infectious organisms. However, it becomes detrimental when it is prolonged or attacks self antigens [1]. Stroke and neurodegenerative diseases such as Alzheimer's disease, multiple sclerosis and Parkinson's disease are associated with a chronic inflammatory response [2,3]. After ischemic stroke, the death of ischemic neurons and especially the release of necrotic cell debris triggers inflammation resulting in strong activation of phagocytic cells [1,4]. Over the past two decades, our understanding of the inflammatory response after stroke and in other diseases has increased due to extensive research. Previously, it was thought that the inflammatory response in brain was beneficial and necessary for repair. Later, it became clear that this "neuro" inflammatory response could be detrimental too, and that even peripheral immune responses can be regulated by the brain [5]. Furthermore, injury to the brain can make the body more vulnerable to systemic infections. For example, a central nervous system injury-induced immunodepression syndrome has been identified in experimental stroke models leading to spontaneous systemic bacterial infections within 3 days after stroke [6-8].

## Stroke

Stroke is a broad term that includes conditions caused by occlusion of or hemorrhage from a blood vessel supplying the brain [7,9]. Its incidence remains high and the number of approved therapies low. As our society ages, the number of stroke patients continues to increase, and will become an important socio-economic burden, as 80% of patients who survive their stroke remain permanently disabled. Ischemic strokes represent more than 80% of all cases and are characterized by the occlusion of a blood vessel due to a thrombus or embolus [10,11]. The location and the size of the ischemic area of the brain varies, depending on which artery is occluded, thereby causing metabolic and functional dysregulations [12,13]. Furthermore, the occlusion can be permanent or transient, the latter meaning that reperfusion will occur. After ischemic stroke, two main regions of damage can be defined according to the remaining blood supply. The area in the brain where complete abolishment of blood supply occurred (blood flow reduced to less than 12 ml/100g/min), is called the core of the insult. Complete, or almost complete, energetic failure resulting in necrosis, defines this region. The penumbra is the area surrounding the core which is hypoperfused during the occlusion period. Collateral blood supply from surrounding arteries ensures that a flow of approximately 30 ml/100g/min is maintained. Some energetic metabolism persists in this region and if reperfusion can be restored quickly, this tissue can be salvaged [14-17]. Consequently, the penumbra is an attractive target to rescue brain tissue as this region can remain potentially viable for 16 to 48 hours, enabling clinicians to intervene and reduce post-stroke disability [7,15].

There are excellent reviews elaborating extensively on the complex ischemic cascade after stroke [5,7,14,17,18]. Briefly and much simplified, three phases can be characterized in infarct progression. The acute phase starts within minutes to a few hours after stroke onset in which the decrease in cerebral blood flow perturbs the ionic homeostasis. This leads to increased intracellular calcium concentrations and stimulation of glutamate release, causing excitotoxicity and a spreading depression throughout the ischemic region [14,18,19]. Water shifts to the intracellular space due to osmotic gradients and cells swell. The resulting vasogenic edema can influence reperfusion negatively and causes intracranial pressure, vascular compression and herniation [18]. Furthermore, generation of reactive oxygen species (ROS), especially if reperfusion takes place, can damage membranes, mitochondria and DNA, leading to misfolding of proteins and enzyme dysfunctions. In the second, subacute phase (a few hours to a few days after ischemia), an apoptotic and neuroinflammatory response develops as a result of the stimulatory influences of the acute phase [14,17,18]. The high intracellular calcium concentrations built up during the acute phase lead to an overactivation of several proteolytic enzyme systems. Finally, in the chronic phase, which can last up to some months after the actual ischemic stroke, repair and regeneration will determine the ultimate extent of damage [19]. As contradictory as it may appear, reperfusion following an occlusion may exacerbate neuronal injury through increased production of ROS and a stronger inflammatory response [16,20]. On the other hand, quickly re-establishing the blood supply is vital as it may allow salvation of neurons in the penumbra from irreversible damage, as energy metabolites and cellular membrane ionic gradients would be restored to normal levels [10]. Recanalization is the only approved and effective therapy for stroke to date. Recombinant tissue plasminogen-activator (rt-PA) must be administered within 4.5 hours after stroke onset to restore reperfusion. Unfortunately, due to the short time window and several risk factors, this treatment can only be administered in 5-10% of cases [11,15]. This emphasizes the need for other neuroprotective therapies not influencing revascularisation, but a strategy that antagonizes, interrupts or slows down injurious biochemical and molecular events in cases treatment with rt-PA is not indicated [21]. Such neuroprotective strategies would primarily focus on reducing the extent of damage in the penumbral region and thus the outcome after stroke [15]. Combining quick reperfusion with the right neuroprotective strategy to address the downstream damaging cascades induced by the occlusion could lead to a better outcome. Nowadays, hypothermia seems to be the most promising neuroprotective therapy that has been translated to clinical practice [22,23]. Many other proven neuroprotectants in experimental models have not improved outcome after ischemic stroke in clinical settings. Several reasons may explain this discrepancy. Firstly, research is performed in many different stroke models ranging from permanent occlusion models to transient ones. Furthermore, the duration of transient occlusion can range between 30 minutes to 3 hours. Therefore, as functional and structural damage after ischemia depends on its severity and duration, it is important to stress which experimental model was used [9]. Secondly, experiments are generally performed in healthy young rodents. But in reality, most patients who suffer stroke have several risk factors (e.g. age, diabetes and hypertension) which negatively interfere with the ischemic cascade. Thirdly, the adaptation mechanism of high phylogenic species such as human beings is expected to be different from lower phylogenic ones. Most experimental approaches involve occlusion of the middle cerebral artery (MCA), the same artery that is most commonly occluded in clinical stroke [11]. In order to improve the relevance of preclinical studies to clinical practice, STAIR (Stroke Therapy Academic Industry Roundtable) criteria were set up [10,22]. Neuroprotective agents that target the acute effects of the ischemic cascade (e.g. glutamate release) do not produce any significant improvement in outcome in a clinical setting, partly due to the (too) short time window to start such therapies. Modulation of later events, like inflammation, could lead to successful neuroprotective therapy [18]. However, in clinical trials, anti-inflammatory drugs have not yet shown the expected results [20,21]. It is hypothesized that such discrepancies indicate that the neuroinflammatory response after stroke is not entirely detrimental and that, without this response, the damage would be even greater. Inflammation causes cell death acutely, but in a later stage, it may help restore body balance [24,25]. Or, vice versa, chronic inflammation can cause tissue damage as well. Therefore, it is necessary to understand the detrimental and neuroprotective effects of each component of the neuroinflammatory response at different time points after ischemic stroke. This will allow a more specific approach to develop therapies targeting different aspects of neuroinflammation (see Table 1).

**Table 1:** The neurotoxic and neuroprotective properties of inflammatory players after ischemic stroke*

*Inflammatory mediators*		Neurotoxicity	Neuroprotection	Effects of hypothermia
1. Cytokines	IL-1β	Endogenous pyrogen Promotion gliosis Increase neurotoxic mediators Increase Ca^2+ ^in neurons Edema formation BBB breakdown Priming endothelium for leukocyte adherence	Increase survival promoting factors Induction of IL-1ra	Reduction increased levels
	TNF-α	Inhibition glutamate uptake Promotion gliosis Increase neurotoxic mediators Increase Ca^2+ ^signaling in neurons Stimulation apoptosis of endothelial cells Edema formation BBB breakdown Priming endothelium for leukocyte adherence Increase NF-κB activation	Increase neurotrophic factors Control extracellular Ca^2+^ Mediation neuronal plasticity Activation repair processes of cerebral microvasculature Induction anti-apoptotic factors Induction anti-oxidants Ischemic tolerance induction	Reduction TNF-α levels Less expression TNF receptor 1 Less NF-κB activation *Reflection:* *Varying properties of soluble and membrane-bound form* *Region-specific concentration and action* *Receptor-specific action*
	IL-6	Endogenous pyrogen Attraction T lymphocytes	Induction IL-1ra	Reduction IL-6
	TGF-β	Increase β-amyloid precursor Increase glial scar formation	Reduction gliosis Less inflammatory mediators Suppressed release ROS Less brain edema Inhibition neutrophil adherence Reduction apoptosis Induction IL-1ra Promotion angiogenesis	*Reflection:* *Neuroprotection limited to penumbra*
	IL-10		Less release cytokines and expression receptors Attenuation astrocytic activation	
2. HMGB Family	HMGB1	Stimulation inflammatory mediators Activation microglia Increase NF-κB activation		Reduction of brain and plasma HMGB1 Reduction of NF-κB activation
3. Chemokines	CINC, MCP-1, MIP-1, MRF-1, fractalkine	Regulation and migration of leukocyte trafficking Stimulation BBB permeability Stimulation phagocytosis Increase cytokine secretion Stimulation apoptosis	Scavenge and repair necrotic tissue Angiogenesis	Downregulation MCP-1
4. Free oxygen radicals	ROS, NO	Lipid peroxidation Stimulation inflammatory response Disruption protein biochemistry		Suppressed oxidative stress
	NO	Induction iron loss of cells Inhibition enzymes DNA replication Stimulation expression inflammatory mediators	Vasodilator	Reduction nNOS No influence iNOS *Reflection:* *Different response in acute and chronic phase*
5. MMPs	MMP-9 (and -2)	BBB breakdown Stimulation leukocyte adherence and transmigration Vasogenic edema Hemorrhagic transformation	Stimulation plasticity, recovery and repair Clearance necrotic cell debris	Reduction MMP-9 No effect on MMP-2 *Reflection:* *Contribution to recovery late after injury*
***Adhesion molecules***				
1. Selectins	E- and P-selectin	Slow down neutrophils and monocytes Promotion rolling over endothelium		Inhibition E-selectin
	P-selectin	Enhancement platelet binding to neutrophils and monocytes		
	L-selectin	Guide unstimulated leukocytes		No effect on L-selectin
2. CAMs	ICAM-1and2, VCAM-1	Stronger attachment leukocytes to endothelium Stimulation diapedesis		
3. Integrins	LFA-1, Mac-1, CD11c	Stimulation adhesion to endothelium Stimulation conformational changes leukocytes for diapedesis		No effect on LFA-1 Delayed upregulation of Mac-1 and CD11c
***Cellular inflammatory response***				
1. Glia	Microglia	Phagocytosis to clear dead cells Production inflammatory and cytotoxic mediators	Production neurotrophic factors Facilitation neurogenesis and plasticity Less release toxic mediators Scavenge and removal necrotic debris	Reduction of activation *Reflection:* *Different subsets have different roles*
	Astrocytes	Production inflammatory and cytotoxic mediators Production chemokines Formation glial scar tissue Enhancement oxidative stress Release glutamate	Production neurotrophic factors Glial scar isolates damaged tissue	
2. Endothelial cells (BBB)		BBB breakdown: Hyperpermeability to macromolecules Vasogenic edema Increase intracranial pressure Stimulation inflammatory mediators and adhesion molecules Astrocyte detachment		Reduction BBB disruption for large molecules
3. Leukocytes	Neutrophils	Release pro-inflammatory and cytotoxic mediators Stimulation lipid peroxidation Release proteolytic enzymes Damage endothelial cell membrane Increase BBB permeability Post-ischemic edema No-reflow phenomenon		Less infiltration
	Monocytes	Generation superoxide anions Release pro-inflammatory cytokines	Removal necrotic cell debris and neutrophils	Less infiltration

* See text for references

## The neuroinflammatory response after ischemic stroke

### General

**Figure 1:**
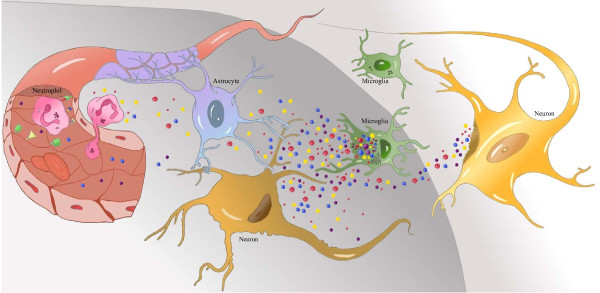
**Schematic overview of the neuroinflammatory response after ischemic stroke**. Microglia become activated after ischemia (grey area) and release pro- and anti-inflammatory mediators. Astrocytes are activated as well and will neglect the maintenance of the neurons, which are most vulnerable to ischemia, and produce neurotoxic and neurotrophic factors. In the ischemic core, neurons die due to necrosis and release necrotic debris into the ischemic tissue, thereby stimulating further activation of glial cells. Astrocytes, together with the attachment of astrocyte endfeet to endothelium and connection with neurons define the neurovascular unit. Neutrophils roll onto the endothelial surface (which is primed by pro-inflammatory cytokines (blue and purple)) until they have slowed down to such a degree that they stick to the endothelium. After binding of selectins to sialyl-Lewis^x ^and CAMs to integrins, the neutrophils undergo conformational changes and flatten. Subsequently, the neutrophils crawl on the endothelium to find an intercellular junction between the endothelial cells for extravasation to the abluminal side and transmigration to the ischemic tissue under the influence of chemokines (red and yellow). Adapted from [26].

Cerebral ischemia leads to the activation of microglia and astrocytes and subsequent production of inflammatory mediators. Such molecules will increase the vulnerability of neurons, cause blood-brain barrier (BBB) disruption and further stimulate gliosis. Consequently, more cell damage and death will occur [7,26,27]. Furthermore, cytokines stimulate the expression of adhesion molecules, mediating the adherence and extravasation of neutrophils and monocytes into the ischemic tissue [26]. Local production of chemokines attracts (via chemotaxis) extravasated leukocytes to the ischemic tissue (Figure 1) [26,28,29].

### Inflammatory mediators

#### Cytokines

Cytokines represent a family of pleiotropic polypeptides (8-26 kDa) that regulate cell activation, proliferation and differentiation [30,31]. Normally, cytokines in the brain are hardly detectable. Their receptors are constitutively expressed, albeit at very low levels. After brain injury, pro- and anti-inflammatory cytokines are quickly and extensively upregulated [26,27,32]. However, the spatial and temporal upregulation of cytokines and their receptors depends on the ischemic model used [33]. Pro-inflammatory cytokines stimulate and aggravate the inflammatory response. The most prominent ones after stroke are interleukin-1β (IL-1β), tumor necrosis factor-α (TNF-α) and IL-6 [17,27,33]. On the other hand, anti-inflammatory cytokines inhibit the expression of pro-inflammatory cytokines and reduce inflammation. Transforming growth factor-β (TGF-β) and IL-10 belong to this category and are most studied after ischemic stroke [27,34]. Other cytokines contribute to tissue damage or repair, but with less pronounced effects than those mentioned. However, cytokines cannot unequivocally be divided into pro- or anti-inflammatory cytokines and may exert neurotoxic as well as neuroprotective effects [12,30,34]. The balance between deleterious and beneficial effects of cytokines will depend on the physiological and biochemical context in the brain [34].

##### IL-1β

A biphasic release pattern has been established for this cytokine in transient as well as permanent MCA occlusion (MCAo) models with a first peak 1 hour after reperfusion and a second peak after 6-24 hours [27,32,35]. Activated microglia account for the larger part of the early production of IL-1β, followed by astrocytes, neurons and endothelial cells [36]. Late expression is due to an influx of inflammatory cells into the central nervous system [35,37]. IL-1β is an endogenous pyrogen which centrally will contribute to an exacerbation of neuronal loss [1]. In the central nervous system, IL-1β stimulates its own production and the expression of other pro-inflammatory mediators such as cytokines and adhesion molecules. Secondly, IL-1β will contribute to the activation and proliferation of microglia and astrocytes. Such a reaction will lead to the upregulation of genes encoding for more neurotoxic mediators, creating a spellbound damaging cycle. Thirdly, IL-1β will stimulate an influx of calcium into neurons, thereby increasing their vulnerability to ischemia. Finally, IL-1β induces edema formation and primes the endothelium for leukocyte adherence [1,12,37-39]. But besides all these neurotoxic effects, even IL-1β can contribute to tissue salvation. IL-1β stimulates the activation of astrocytes, which in their turn will produce survival-promoting factors [1,39]. Moreover, increases in IL-1β will lead to increased levels of IL-1ra, an antagonist of IL-1β. The temporal induction profile of IL-1ra after ischemia virtually parallels that of IL-1β which may suggest that the balance between IL-1β and IL-1ra is more important than the levels of IL-1β itself [18,39]. In permanent and transient MCAo models, administration of IL-1ra reduces tissue damage significantly [40-42]. Even if the administration is delayed up to 3 hours after a 1-hour MCAo, rats show a reduction of 60% in cortical lesion volume [43]. A clinical phase II safety trial performed in acute stroke patients with the recombinant methionylated form of human Il-1ra (Anakinra or Kineret^®^) proved the product to be safe and improved functional outcome [39,44]. Recently, a dose-ranging study has been performed in stroke patients to assess if Anakinra can easily cross the BBB and reach effective concentrations when administered intravenously. The results were favorable and showed that IL-1ra can enter the CSF and that the rate of entry can be modulated by altering the administration regime [45]. If the right therapeutic window can now be determined in patients, this agent might be a promising clinical effective neuroprotective agent after stroke.

##### TNF-α

Contrary to IL-1β, TNF-α is a more pleiotropic cytokine with neurotoxic and neuroprotective effects [46]. TNF-α also shows a biphasic release pattern with a first peak 1-3 hours and a second peak 24-36 hours after an ischemic insult [27,32,47]. However, it is important to make a distinction between soluble and membrane-bound TNF-α [48]. Activated microglia and macrophages are major producers of soluble TNF-α within the first 6 hours after cerebral ischemia [49-51]. Although IL-1β and TNF-α often work synergistically, they are produced by non-overlapping subsets of microglia/macrophages. A maximum of 1.2% of microglia and 4.5% of macrophages co-express IL-1β and TNF-α within the first day after a permanent MCAo [52]. Furthermore, TNF-α may show higher production rates in certain regions (e.g. striatum) and, depending on the region, TNF-α can either be detrimental or beneficial. For example, TNF-α released in the striatum is considered to cause neurodegeneration, while release in the hippocampus could promote neuroprotection [30]. TNF-α can potentiate excitotoxicity *in vitro *by inhibiting glutamate uptake [30,53,54]. It also activates glia, thereby promoting its own production and that of other neurotoxic mediators, leading to more oxidative outburst [50,53]. Such activation also boosts the production of neurotrophic factors such as BDNF, GDNF and VEGF. Furthermore, TNF-α can increase neuronal vulnerability by stimulating intraneuronal calcium signaling *in vitro *[55]. TNF-α also controls the (down-) regulation of extracellular calcium in astrocytes, thereby reducing damage [30,56]. TNF-α stimulates apoptosis of endothelial cells and contributes to vasogenic edema and, due to this BBB breakdown, infiltration of circulatory inflammatory cells are stimulated [57]. On the other hand, TNF-α activates repair processes of the cerebral microvasculature and mediates neuronal plasticity [30]. Perhaps most importantly, TNF-α activates the NF-κB pathway that is involved in signaling cell death (apoptosis) as well as cell survival. NF-κB will stimulate the production of pro-inflammatory cytokines [30,53,58]. Ultimately, the balance between the two signals will determine the toxic degree of TNF-α [12,30,54]. Several hypotheses exist. One suggests that the detrimental effects occur in the early phase of the inflammatory response and the more beneficial effects in a later stage [24]. Another hypothesis relates to the receptors to which TNF-α binds. Soluble TNF-α (which binds to TNF receptor 1) would cause primarily detrimental effects whereas membrane bound TNF-α (which binds to TNF receptor 2) would signal for neuroprotection [33,59]. Other studies suggest that TNF-α can also be neuroprotective by acting through TNF receptor 1 [48,50,60]. Whatever the case may be, research shows that inhibition of TNF-α, using antibodies given at the onset of reperfusion, reduces infarct volume after a 2-hour MCAo, but only by 16% [41,47]. In contrast, mice lacking both TNF receptors show increased infarct volume after ischemic stroke [61], and administration of TNF-α 48 hours prior to induction of MCAo results in a neuroprotective effect. Thus, TNF-α can cause ischemic tolerance and may stimulate tissue repair [41]. In conclusion, there is no consensus on the effect of TNF-α after ischemic stroke. Neurotoxic or neuroprotective effects will depend on several factors such as the extent of microglial activation in specific brain regions, timing and threshold of TNF-α expression and of its receptors and on the conditions that stimulate TNF-α signaling [12,30]. It is important to know in which form TNF-α is induced, in which cells, and on which receptor it will act. For instance, TNF-α in neutrophils and endothelial cells stimulate injury whereas in neurons it is neuroprotective [62].

##### IL-6

IL-6 is detected 4 hours after stroke onset, with peak concentrations after a day, and remains detectable for up to 14 days [24,32,63]. Activated microglia, followed by astrocytes, neurons and invading cells of the immune system, comprise the main source for IL-6 [24,35]. Like IL-1β, IL-6 is an endogenous pyrogen. It helps to attract T-lymphocytes to the brain and will in general contribute to an exacerbation of the inflammatory response. However, IL-6 can upregulate IL-1ra, and IL-6-deficient mice do not show improved outcome after stroke [12,26,64]. In clinical studies, IL-6 is often suggested as a good marker to predict the severity of an insult as high plasma levels correlate with the severity of the stroke [65,66].

##### Anti-inflammatory cytokines

Only two of the five different *TGF-β *cytokines are prominent after stroke; these peak 6 hours after the onset of a lesion. TGF-β1 is primarily produced by activated microglia and macrophages, but some production is also visible in neurons and T-cells [34,67]. TGF-β2 on the other hand is produced by astrocytes and neurons [68]. Generally, TGF-β controls cellular processes like proliferation, differentiation, apoptosis and migration [63,69]. More specifically after stroke, it reduces glial activation, decreases the expression and efficacy of other cytokines, suppresses the release of harmful oxygen and nitrogen derived products, promotes angiogenesis in the penumbral area, causes less brain edema with less neutrophil adherence to endothelial cells, and stimulates the release of IL-1ra [41,70]. However, as TGF-β1 can inhibit apoptosis of neurons, but not necrosis, its possible protective influence is consequently limited to the penumbra. On the other hand, TGF-β1 stimulates glial scar formation and production of beta amyloid precursor, which can lead to a higher risk of cognitive deficit. Although TGF-β works on several inflammatory pathways simultaneously to protect the brain, the endogenous concentration is too low to inhibit serious damage after stroke. Administration of TGF-β before the induction of an ischemic insult rescues neurons from cell death [33,69,71].

*IL-10 *is another constitutively expressed anti-inflammatory cytokine with peak levels 3 days after stroke onset. The primary source is activated microglia and astrocytes. IL-10 inhibits cytokine production and the expression of their receptors and attenuates astrocytic activation. IL-10 administration can reduce damage after MCAo [12,27,34].

#### High-mobility group box (HMGB) protein family

Neuroinflammation can also be triggered by members of the HMGB protein family, which are related to cytokines. They function as sentinels of cellular stress (e.g. necrosis). HMGB proteins are ubiquitously expressed in many cell types. Specific structures mediate their direct binding to DNA and intracellular proteins (e.g. histones and transcription factors) to regulate, among others, DNA structure, repair and transcription [72,73]. HMGB1 is released in response to pathogen-associated molecular patterns or cellular stress (e.g. stroke) and functions as a pro-inflammatory cytokine [74,75]. It translocates from the nucleus to the cytoplasm or even completely disappears from the cells within 1 hour after induction of cerebral ischemia in mice [76,77]. HMGB1 is secreted by neurons and cells of the immune system and passive release also occurs from necrotic cells [77]. Although apoptotic cells release only small amounts of HMGB1, the release of this compound by phagocytosis of apoptotic cells cannot be underestimated [78]. The effects of recombinant HMGB1 have been addressed *in vitro *as well as *in vivo*. In glial cell cultures, HMGB1 induces the expression of iNOS, IL-1β and TNF-α; augments excitotoxicity-induced neuronal cell death and induces microglial activation [79]. Microinjection of HMGB1 into the cortex of mice results in increased expression of iNOS and IL-1β [77]. Three cell surface receptors can bind extracellular HMGB1 and activate NF-κB subsequently: Toll-like receptors (TLR) 2 and 4 and the receptor for advanced glycated end products (RAGE). Binding of HMGB1 to RAGE also stimulates chemotaxis [80-82]. A deficiency in these receptors was observed in an MCAo model for cerebral stroke, and this leads to increased infarct size [82]. A role for HMGB1 in cerebral ischemia is indicated by the observation that downregulation of HMGB1 by RNA interference or by using neutralizing antibodies leads to reduced infarct volume and suppression of microglial activation and neuroinflammation in rat and mouse MCAo models [79,83]. In patients who suffer cerebral ischemia, HMGB1 levels in the serum are increased [84].

#### Chemokines

Chemokines are small (8-10 kDa), inducible molecules, structurally and functionally related to cytokines, that stand out because of their chemotactic activity for leukocytes [85-87]. There are 40 different known chemokines thus far, which all share a common structural pattern with 4 cysteine residues, which leads to their classification into 4 subfamilies of which 2 are important after stroke: the C-X-C and C-C family [29,41,88]. The C-X-C family attracts neutrophils and the C-C family monocytes/macrophages [41,85]. Constitutively, chemokines and their receptors are expressed in very low concentrations [29,86]. Cytokines (especially IL-1β and TNF-α) stimulate the production and release of specific chemokines after cerebral ischemia, such as CINC (cytokine induced neutrophil chemoattractant), MCP-1 (monocyte chemoattractant 1), fractalkine, MRF-1 (microglial response factor-1) and MIP-1 (macrophage inflammatory protein 1). These are upregulated in the first 3 hours after ischemic stroke and remain high for at least 6 hours [29]. The earliest part of the production is ascribed to activated microglia, followed by astrocytes and injured neurons [27,28]. However, depending on cell type, other chemokines are upregulated. While neurons express fractalkine and MCP-1 after cerebral ischemia, microglia show increased expression of MIP-1-α, MIP-2 and MRF-1 [33]. Furthermore, when reperfusion is established, other chemokines, like IL-8, are upregulated as well [26,89]. Under normal conditions, chemokines control the positioning of cells in tissues and recruit leukocytes to site of inflammation [87]. After brain injury, chemokines serve as signals released into the extracellular fluid and cerebrospinal fluid to recruit microglia, neutrophils and monocytes (chemotaxis) [7,29,33]. To achieve leukocyte recruitment, chemokines co-operate with adhesion molecules and affect BBB permeability to ensure diapedesis through the vessel wall [27-29]. Furthermore, chemokines stimulate apoptosis and phagocytosis [28].

IL-8 is a major chemotactic cytokine in humans, but has not been identified in rodents, only its equivalent CINC. Inhibition of CINC, using antibodies, reduces infarct size in a transient MCAo model when given before (1 day) and upon reperfusion [89]. Blocking the activity of MCP-1 with antibodies or MCP-1 deficiency in mice reduces infarct size in transient and permanent MCAo models [90,91]. Inhibiting the activity of MIP-3-α reduces infarct size after transient MCAo [92]. Fractalkine-deficient mice provide neuroprotection in transient (30 minutes to 2 hours) MCAo models [33,93,94]. In general, transgenic mice overexpressing chemokines show chronic neutrophil infiltration, continuous glial activation and BBB disruption. They ultimately die by a terminal wasting syndrome. Chemokine knock-out mice, on the other hand, show deficiency in leukocyte recruitment [29]. Therapy with a broad spectrum pan-chemokine inhibitor, given at the onset of reperfusion, can reduce infarct volume by 50% after a 1-hour MCAo in rats [95]. These animals showed less macrophage accumulation in the peri-infarct area, but not in the core of the insult. This supports the hypothesis that inflammatory cells contribute to extended damage to the penumbra [95].

#### Free oxygen radicals

Oxidative stress can damage the organism if the physiological balance between oxidants and anti-oxidants is disrupted in favor of the former. Oxidative stress is induced after cerebral ischemia by various pathways of the ischemic cascade; especially inflammation and reperfusion increase the production of ROS [7]. The key radical after stroke is superoxide anion, produced by xanthine oxidase and NADPH oxidase. OH• radicals are increased 2 hours after stroke onset [96]. L-arginin is transformed into nitric oxide (NO) via 3 types of NO synthases: neuronal, endothelial and inducible (n-, e-, iNOS respectively). These NOS are increased after brain ischemia [7]. In rodents, eNOS is upregulated directly whereas nNOS is upregulated a day after ischemia onset and iNOS even later. In the core of the insult, production is seen in neutrophils and macrophages, while at the margins of the infarct it is seen in microglia, blood vessels and astrocytes [38,60,96]. NO has actually a dual role. On the one hand, NO can reduce damage after stroke by helping to restore blood supply to the ischemic area; on the other hand, NO can form radicals by reacting, primarily with superoxide anion radical or other molecules with free electrons, to form peroxynitrite [33]. These radicals contribute to lipid peroxidation and protein biochemistry disruption by causing an imbalance between signal transduction mechanisms and cellular toxicity [33]. Furthermore, NO can stimulate the expression of inflammatory mediators and adhesion molecules, induce iron loss from cells, and inhibit enzymes for DNA replication (causing DNA damage) [97]. Which of these contradictory effects will prevail depends on the concentration of NO, the sensitivity of the cell type to NO and the difference between the acute or chronic phase of inflammation [98]. Knock-out mice for nNOS show reduced infarct volumes whereas in knock-out mice for eNOS the injury after ischemic stroke is increased [62,97]. This suggests that the early production of NO from nNOS leads to tissue damage while NO from eNOS has a protective function by dilating the vessels and thereby regulating blood flow to the penumbra [99]. Depletion of iNOS has no effect on infarct volume after 1 day, but it should be noted that the increase in iNOS occurs at later time points [62,96,97].

#### Matrix metalloproteinases (MMPs)

MMPs are a group of at least 28 zinc-dependent endopeptidases that can proteolytically degrade a variety of molecules that constitute the extracellular matrix components [100-102]. Physiologically, these proteases are involved in tissue development, wound healing, bone growth, ovulation and angiogenesis. After brain trauma, MMPs are upregulated and activated after cleavage of their pro-forms under influence of inflammatory mediators [103,104]. After a transient MCAo, MMP-2 (gelatinase A) and MMP-9 (gelatinase-B) appear within 3 hours in the core and stay detectable up to 7 days [16,100,101]. However, there are studies that report no obvious increase of MMP-2 after ischemic stroke compared to the observed constitutive expression [24,38,62,104,105]. If MMP-2 is upregulated, it peaks later. MMP-9 is first produced by endothelial cells and neutrophils and later predominantly by macrophages (after 5 days) [106]. Indeed, neutrophils contain and degranulate gelatinase granules resulting in release of pro-MMP-9 and MMP-9 within a day after stroke onset [107]. Although complete neutropenia can reduce the increased levels of MMP-9 after transient MCAo, it cannot entirely abolish the upregulation after ischemic stroke [108]. MMP-9 expression by endothelial cells within and at the periphery of the ischemic lesion is said to be the key mechanism by which the endothelial wall is compromised [106]. The fact that MMP-2 and -9 degrade type IV collagen, laminin and fibronectin, which are major components of the basal lamina around cerebral blood vessels and the extracellular matrix, will certainly underpin this theory [10,38,101,106]. This digestion starts as early as 2 hours after ischemia which correlates with BBB breakdown 3 hours after ischemia [16]. In patients, a correlation could be made between the biphasic opening pattern of the BBB and the involvement of MMPs. In the early reversible opening (after 3 hours), there is an increase in plasma MMP-2. One to 2 days after ischemia, the severe and late opening of the BBB correlates with increased MMP-9 [109]. However, even such destructive molecules have some good qualities. It has been noticed that 7 days post-ischemia, MMPs are involved in plasticity, recovery and repair [109]. The late increase may aid in the migration of macrophages into the ischemic lesion and contribute to clearing of cellular debris [106,109]. Broad-spectrum inhibitors of MMPs, such as BB-94 and KB-R7785, administered after stroke onset, reduce damage after permanent MCAo in mice by 26% [100,101]. This is also combined with reduced BBB opening [110]. Such treatments however, cause serious side effects due to their low specificity and explain why these inhibitors have not been used in clinical practice [47,103]. On that account, more selective inhibitors or knock-out mice for MMP-2 or -9 have been explored. MMP-2 knock-out mice subjected to 2 hours occlusion show massive upregulation of MMP-9 and obviously no improvement could be observed [111]. MMP-9 knock-out mice, on the other hand, do show better outcome after ischemic stroke [107,112,113], and inhibition of MMP-9 as late as 4 hours after ischemia onset decreases infarct size [16,106]. A selective inhibitor for MMP-2 and -9 (SB-3CT) in mice subjected to a 2-hour MCAo reduces lesion size up to 6 hours after ischemia onset, and this inhibitor is well tolerated in animals [103]. Additionally, MMP levels in plasma could be a good biomarker to predict the severity of stroke. In rats, levels of pro-MMP-9 and MMP-9 correlate with infarct volume [105].

### Adhesion molecules

Adhesion molecules comprise three groups of molecules that all serve one purpose: adherence and extravasation of neutrophils and monocytes out of the blood vessel, through the BBB into the ischemic tissue. Therefore, some necessary bindings must occur: first selectins bind to neutrophils and integrin receptors on neutrophils bind to cellular adhesion molecules (CAMs) on the endothelial vessel wall [7,26,27].

#### Selectins

Selectins are membrane-bound glycoproteins [26]. There are three selectins, characterized by a common sequence and structural features, E- (endothelial), P- (platelet) and L- (leukocyte) selectin [114]. Selectins bind to carbohydrate residues (sialyl-Lewis^x^) on neutrophils and monocytes, thereby promoting a first binding to the endothelium which is necessary to slow down neutrophils and monocytes and promote rolling over the endothelium during the early stage of activation [26]. E-selectin is produced by stimulated endothelium and leukocytes and P-selectin also by platelets, hence the name. Stimulation occurs via cytokines and other inflammatory mediators. Four hours after the onset of stroke and up to 70 hours after reperfusion, increased levels of E-selectin are noticed [26]. There is a constitutive expression of P-selectin, although basal levels almost escape routine detection. Due to preformed pools of P-selectin in endothelial cells and platelets, P-selectin can be upregulated quickly and faster than E-selectin after stroke [26,115]. Looking at the temporal profile of P-selectin, a biphasic release pattern can be distinguished with a first peak 15 minutes after the insult. This then falls back to basal levels within 1 hour after the insult. The second peak, at 6 hours after reperfusion in transient MCAo models, is ascribed to new synthesis of P-selectin protein [116,117]. Plasma levels of P-selectin are often determined to assess the severity of an insult. This is possible as there are actually 2 isoforms, one with and one without a transmembrane region. The former is expressed on the cell surface and will only be shed after proteolysis while the latter is secreted directly into the circulation from the preformed pools [115]. Inhibiting the activity of P-selectin alone by treatment with monoclonal antibodies (ARP 2-4, RMP-1) after the onset of the insult, does not reduce the infarct volume significantly [26,118-120]. This suggests that the involvement of P-selectin in the inflammatory response after ischemic injury starts early. The exact function of L-selectin is not known. It is believed that it guides unstimulated leukocytes to the endothelium to facilitate transmigration [26,27]. However, post-treatment with inhibitors of L-selectin using monoclonal antibodies shows no improvement of lesion size in rabbits subjected to 2 hours occlusion [121]. Inhibition of the binding of selectins to their ligands by administration of a synthetic analogue of sialyl-Lewis^x ^reduced infarct volume after a transient MCAo, even if it was given as late as 2 hours after onset of the insult [119,122].

#### Cellular adhesion molecules (CAMs)

The CAMs belong to the immunoglobulin superfamily. After stroke, intracellular adhesion molecule (ICAM) -1 and -2, vascular adhesion molecule (VCAM)-1 and platelet endothelial cell adhesion molecule (PECAM)-1 will contribute to the inflammatory response by attaching neutrophils and monocytes more tightly to the endothelial wall for facilitating and even stimulating diapedesis through the vessel wall to the site of injury [26,27,123]. These CAMs are constitutively expressed on cell membrane of endothelial cells, leukocytes, epithelial cells and fibroblasts. Furthermore, higher levels are observed in hypertensive than in normotensive rats [41]. After trauma, inducible ICAM-1 is quickly upregulated, especially in the core of the insult [62]. The increases in ICAM-1 and VCAM-1 after stroke are influenced by IL-1β and TNF-α [26,27,124]. ICAM-1 levels peak 12 hours after stroke onset and are maintained during 24 hours whereas VCAM-1 only peaks after a day in a 1-hour MCAo model [24,27,123,124]. Soluble isoforms of these adhesion molecules can be quantified in peripheral blood after they have been shed from the surface of activated cells. In patients, VCAM-1 was increased up to 5 days after symptom onset while ICAM-2 and PECAM-1 were not increased after injury [123,124]. As these polypeptides contribute to aggravation of damage after stroke, antibodies for these CAMs were evaluated. Administration of a murine anti-ICAM-1 antibody (enlimomab) after a 2-hour MCAo resulted in a decrease in infarct volume of 44% [125,126]. However, in permanent occlusion models there was no such positive influence of this treatment. Similar results were seen with antisense oligonucleotides for ICAM-1 [127]. These data suggest that there is a need for reperfusion for activation of the inflammatory response [126]. An anti-VCAM-1 monoclonal antibody was tested too, but showed no reduction in infarct size after transient cerebral ischemia in rodents [128]. In a phase III clinical trial, enlimomab was given within 6 hours after symptom onset in cerebral ischemia and maintained over 4 days. Unfortunately, the treated group showed a 43% higher mortality, possibly due to the murine origin of the antibody [20,124,129]. Surprisingly, for PECAM-1, there is discussion about possible neuroprotective properties aside from the neurotoxic ones. PECAM-1 knock-out mice show facilitated leukocyte transendothelial migration after histamine treatment which contradicts the current hypothesis that PECAM-1 stimulates migration [123].

#### Integrins

CAMs bind to integrins on neutrophils and monocytes. After stroke, CD18 or β2 integrins are activated; these can be further divided into 3 different receptors with the same β-chain, but 3 distinct different α-chains, CD11a/CD18 (or LFA-1), CD11b/CD18 (or Mac-1) and CD11c/CD18. The first one primarily binds to ICAM-1 and -2, the second to ICAM-1 and the third to complement fragments [20,124,130]. LFA-1 or lymphocyte function-associated antigen-1 is expressed on peripheral blood lymphocytes [131]. Mac-1 or the leukocyte adhesion receptor macrophage-1 antigen is constitutively expressed on leukocytes and is transformed into an activated conformation as well as quantitatively upregulated on the endothelial cell surface after ischemia [130]. Physiologically, integrins connect endothelial cells to components of the underlying basal lamina [41,132]. CD18 integrin production can be influenced by cytokines (especially TNF-α), chemokines and the adhesion of neutrophils to E-selectin [26,124,133]. Due to upregulation of LFA-1 and Mac-1 after stroke, more neutrophils will adhere to the vessel wall and undergo the necessary conformational changes to cross the BBB [133]. Therefore, research has focused on these integrins as potential targets for stroke therapy. Mice with null mutations for LFA-1, subjected to 1-hour occlusion, showed less leukocyte and platelet recruitment compared to their wild types [129]. Mac-1-deficient mice or treatment with monoclonal antibodies showed reduced infarct size after a transient occlusion of 3 hours in mice, but this coincided with serious side effects including peripheral white blood cell depletion [20,130]. A humanized anti-Mac-1 antibody (called Leukarrest) was evaluated in a phase III clinical trial in which the agent was administered up to 12 hours after symptom onset. The trial showed no improvement in treated subjects, possibly due to the large time-window for treatment onset [20]. Research was also performed with a humanized monoclonal antibody to all CD11/CD18 integrins. In rabbits, post-treatment with this antibody after a 2-hour occlusion attenuates infarct size after the insult [20,134]. However, no protection was observed in permanent occlusion models [20,135].

### Cellular inflammatory response

There is a hierarchy of vulnerability to ischemia. Unfortunately, neurons are the most vulnerable, followed by astrocytes, microglia and endothelial cells [38,136]. Not only are other cells less vulnerable, they will also participate in and aggravate the inflammatory response, thereby inducing mostly deleterious but also some beneficial effects [7,26,27].

#### Glia

Glial cells (microglia and astrocytes) are necessary to support the central nervous system.

*Microglia *represent 5-20% of the total glial population and are key modulators of the immune response in the brain [137,138]. Their main characteristic is their sensitivity to changes. Quiescent resting microglia show a ramified state characterized by a small cell soma and extensive branches projecting out of the cell body [2,33,139]. When a foreign aggressor occurs, microglia change from this monitoring state to one of protection and repair, characterized by an amoeboid form very similar to macrophages and are therefore called "resident" macrophages. Using phagocytosis, microglia will clear foreign organisms [27,33,137-140]. However, after ischemia, this immune response turns on its own cells, becomes destructive and will lead to cell damage. How microglia become activated after ischemic stroke is still not clear. A possible mechanism is rupture of necrotic neurons in the core of the insult, leading to release of their contents into the extracellular space and scavenging of these contents by microglia [4,141]. ROS play a major part in the activation and proliferation of microglia [27]. The penumbra in particular is vulnerable to microglial activation where this causes considerable secondary cell death [33]. Resident macrophages are detected as soon as 2 hours after ischemia onset, whereas blood-born macrophages do not enter the brain before 10 hours. At 22-46 hours, blood-born as well as resident macrophages are distributed over the entire lesion and stay detectable up to 1 week after the insult [13,14,32,52,137]. Microglia in the core of the insult survive the period of reduced blood flow if it does not exceed 90 minutes of occlusion [142]. Nevertheless, microglia/macrophages surrounding the ischemic tissue will migrate toward the ischemic lesion and engage in close contact with neurons ("capping"). As these neurons die later on, this capping ensures early recognition and fast phagocytic removal of dying/dead neurons [139,142]. Surprisingly, defective expression of LFA-1 eliminates the capacity of microglial cells to migrate toward injured neurons [131]. In an activated state, microglia will produce inflammatory and cytotoxic mediators contributing to cell damage and cell death. On the other hand, microglia are a major producer of TGF-β1 which supports the hypothesis that microglial activation is also neuroprotective [33]. There are some possible mechanisms supporting this theory. First of all, microglia produce neurotrophic factors which stimulate neurogenesis and plasticity. Secondly, phagocytosis of neutrophils by activated microglia in the lesion prevents the release of extra toxic mediators [2,143]. Finally, resident macrophages scavenge and remove necrotic debris and harmful components. Indeed, these data suggest that early activation is detrimental and later activation beneficial [2]. Different subsets of microglia may have different roles after ischemic stroke and thus improve or reduce the chances of survival of ischemic neurons [144]. Perhaps an ideal therapy should modulate the microglial response in order to stimulate neurogenesis [145]. Transgenic mice in which microglial proliferation can be inhibited (eliminated) show increased infarct volume (by 13%) after a 1-hour occlusion, which suggests that proliferating resident microglial cells exert a neuroprotective role after ischemia [146].

*Astrocytes *are essential for the maintenance of the central nervous system. They too will proliferate and differentiate (astrogliosis) after stroke, which coincides with increased production of glial fibrillary acidic protein (GFAP). Astrogliosis can be potentially destructive after an insult [12,27]. A massive astroglial response, starting in the core of the lesion 4 hours after the trauma, is observed up to 28 days after stroke onset in the photothrombosis model [147]. However, 10 minutes of occlusion results in astrogliosis only after 1 day with peak activity 4 days after the insult [69]. Astrocytes supply energy to neurons, produce precursors of neurotransmitters, form an anti-oxidative defense and secrete neuroprotective and neurogenic factors. Their end-feet form, together with the endothelial cells, the BBB and thereby they contribute to the maintenance of the ionic homeostasis [38,147,148]. Astrocytic activation leads to the production of inflammatory mediators and cytotoxic molecules (ROS, NOS, proteases, etc.) [138,147]. They express high levels of chemokines and chemokine receptors, but it is not proven that astrocytes can migrate toward these factors [86]. Glial scar tissue will replace damaged tissue which has neurotoxic as well as neuroprotective effects. On the one hand, this barrier will prevent axonal ingrowth and reinnervation, thereby impeding recovery. On the other hand, this barrier will isolate the damaged tissue from viable tissue [147].

#### Endothelial cells and the blood-brain barrier (BBB)

The BBB comprises a ternary structure of endothelial cells with tight junctions distinguishable from the more fenestrated peripheral endothelium, followed by a basal lamina, which is an extension of the extracellular matrix, and finally the end-foot processes of astrocytes [116,149]. The interconnection is mediated by integrins [41]. This BBB is needed to maintain the fragile extracellular microenvironment via a twofold function. On the one hand, it protects sensitive neurons from contact with potentially toxic activated plasma proteases [148,150]. On the other hand, the BBB ensures the supply of nutrients by specific transport systems [148]. To improve understanding of the BBB and its effect on neurons, the hypothesis of a neurovascular unit was suggested. This consists of microvessels (with the above mentioned ternary structure) that communicate with astrocytes which in turn are connected to neurons and their axons. Microglia contribute to the integrity of the neurovascular unit. Adjacent neurovascular units communicate with each other [38,102]. After ischemia, subtle or dynamical changes in BBB permeability occur and can be transient or permanent depending on the severity of the insult. The latter is characterized by endothelial swelling, astrocyte detachment and blood vessel rupture in the ischemic area while transient BBB disruption shows endothelial hyperpermeability to macromolecules in the peri-infarct area [151]. Transient BBB disruption shows a biphasic pattern with an initial opening 2-3 hours after the onset of the insult, while 24-48 hours after reperfusion a second opening occurs, leading to vasogenic edema and increased intracranial pressure [10,151]. Furthermore, production of pro-inflammatory cytokines and adhesion molecules will be stimulated. Such a disruption results in rapid but significant changes in the molecular relationship between astrocytes and the microvascular extracellular matrix, which has a feedback effect on the neurons they supply and protect [132].

#### Leukocytes

Neutrophils invade ischemic tissue first, followed by monocytes.

Five hours after reperfusion, *neutrophils *are already present in damaged tissue [27,32]. In a permanent MCAo model, neutrophils peak 12 hours after insult onset [63]. Due to increased adhesion molecules after stroke, neutrophils undergo conformational changes (actually modifications in the cellular cytoskeleton) and migrate through the endothelial vessel wall (diapedesis) [26]. After this crossing, chemokines will attract neutrophils to ischemic tissue via a concentration gradient (chemotaxis) [18,26,27,152]. Once there, neutrophils contribute to secondary injury of potentially viable tissue by releasing pro-inflammatory cytokines and other cytotoxic products (proteases, ROS, MMPs) [26,27,89]. The release of proteolytic enzymes damage endothelial cell membranes and basal lamina, increases BBB permeability and contributes to post-ischemic edema. Neutrophil activation can alter cerebral artery vasoreactivity [41]. Moreover, due to their adherence onto the vessel wall, they create a secondary obstruction in the cerebral microvasculature, called the no-reflow phenomenon [18,26,153]. Inhibiting neutrophil infiltration does improve outcome, but only in transient experimental stroke models [27,95]. Four to 6 hours after invasion of neutrophils, *monocytes *adhere to vessel walls as well and migrate into ischemic tissue with peak activity 3-7 days after the onset of the insult [63,154]. These monocytes will replace the neutrophils [20,153]. When activated, they transform into blood-borne macrophages. On the one hand, macrophages generate superoxide anions, secrete pro-inflammatory cytokines and create a pro-thrombotic environment, while on the other hand, phagocytosis will remove necrotic cell debris and neutrophils [20].

## Hypothermia

As stroke is a multi-faceted phenomenon, a neuroprotective approach of choice should act on several levels of the ischemic cascade. With respect to inflammation, toxic effects should be inhibited while the associated repair mechanisms should be stimulated. Moderate (30-32°C) to mild (32-34°C) hypothermia could be a good option, as it acts on several pathways of the ischemic cascade and exerts differential effects on the inflammatory response at different time points [23]. Indeed, data from the literature indicate that stimulating and inhibiting effects may be involved in the neuroprotective effects of hypothermia. In cardiac arrest, hypothermia has already proven to be neuroprotective in a clinical setting [155]. Furthermore, in experimental models of transient cerebral ischemia, moderate-to-mild hypothermia can reduce infarct volume by more than 50% [156,157]. However, in permanent MCAo models the effect is less clear.

Fever occurs often after ischemic stroke and increases cell damage and cell death [158]. If hypothermia is induced, more neurons survive the ischemic period [159]. However, the neuroprotective effect of hypothermia is not yet fully elucidated. Hypothermia has no effect on the cerebral blood flow in the penumbra during ischemia, but can block hyperperfusion after ischemic insult [23,160]. Furthermore, the neuroprotective effect of hypothermia cannot be explained by solely a reduction in cerebral metabolic demand or glutamate release as hypothermia still reduces infarct volume when initiated in the subacute phase of the ischemic cascade, when the initial increase in glutamate has already passed [156,161,162]. Consequently, hypothermia affects several pathways.

Hypothermia has effects on the neuroinflammatory response. Unlike anti-inflammatory drugs, hypothermia exerts both stimulating and inhibiting effects on the inflammatory response (see table 1). Hypothermia reduces the increase of IL-1β after stroke [153,162-164]. It would be interesting to know what a combination of hypothermia with IL-1ra could accomplish. While there is no consensus on the role of TNF-α after stroke, there is also as of yet no consensus on the effect of hypothermia on TNF-α. Most studies show a reduction of TNF-α after hypothermic treatment. More interesting is that mild hypothermia can also influence the expression of TNF-α receptors and the activation of NF-κB [23,165]. Hypothermia can reduce the increased expression of TNF receptor 1 as early as 15 minutes after traumatic brain injury [165]. This receptor could be associated with detrimental effects after brain injury. It would be interesting to know if hypothermia also decreases the expression of TNF receptor 2. A recent study using a transient MCAo model showed that pre- as well as post-ischemic hypothermia is able to attenuate HMGB1 protein levels in brain and plasma [166]. However, little literature exists regarding the influence of hypothermia on the expression of the main receptors of HMGB1. TNF receptors and TLRs are known to primarily mediate activation of NF-κB. Indeed, hypothermia has anti-apoptotic effects that could also be mediated by affecting NF-κB [96,153,157]. In normal conditions, NF-κB is present in the cytoplasm, but is bound to a family of inhibiting proteins. To become activated, IκB kinase must phosphorylate these inhibiting proteins to liberate NF-κB and let it enter the nucleus and induce gene expression [153,167]. In a 2-hour MCAo model, increased NF-κB binding was observed as early as 2 hours after ischemia. Intra- and post-ischemic hypothermia was able to reduce IκB kinase expression, resulting in less NFκB translocation which could be the result of two pathways [153,167,168]: (1) hypothermia may directly influence the expression of IκB kinase, or (2) cells that are protected by hypothermia may influence surrounding cells by releasing fewer inflammatory stimuli [167]. Inhibition of NF-κB activation could be key to the neuroprotective effects of hypothermia, but it should be taken into account that NF-κB also exerts anti-apoptotic effects. Indeed, there are conflicting reports on this subject as both increases and decreases in ischemic injury have been observed when inhibiting the activity of NF-κB [167-169]. Ultimately, it will be the balance between type I and type II TNF-α receptor expression and the signaling pathways involved that will determine the outcome [170]. Chemokines are reduced by hypothermic treatment. Recently, a study showed downregulation of MCP-1 after application of mild hypothermia *in vitro *[92]. However, these results need to be confirmed *in vivo*. Hypothermia can suppress oxidative stress after an insult. Superoxide anion radical production is attenuated by a post-ischemic moderate hypothermic treatment [166]. These results also confirm earlier findings that hypothermia reduces the production of hydroxyl radicals after a transient MCAo in rats [96]. Further research has shown that post- but not intra-ischemic hypothermia reduces the release of superoxide anions, as most ROS are only produced in the reperfusion phase [171]. Concerning NOS, it has been shown that hypothermia decreases nNOS by 50% in the penumbra, while no effect is seen on iNOS [96]. Intra-ischemic mild hypothermia reduces expression of MMP-9 in rats subjected to 2 hours of MCAo [104]. There is no effect on MMP-2, neither after stroke nor after hypothermic treatment up to 24 hours after the insult [104]. Also, MMP-9 expression is low in patients after hypothermia, which could suggest a temperature sensitivity of MMPs [172]. *In vitro *studies show that hypothermia can reversibly inhibit E-selectin, without affecting L-selectin [173,174]. Furthermore, 2 hours of mild post-ischemic hypothermia reduces ICAM-1 levels in brain and plasma for up to 7 days after 2 hours of MCAo [162,166,175]. *In vitro*, as well as in patients with cardiopulmonary bypass, hypothermia influences integrin expression. Hypothermia (27°C) delays upregulation of Mac-1 and CD11c, but has no effect on LFA-1 [174,176]. Hypothermia can inhibit but possibly only delay glial activation [177,178]. Hypothermia appears to decrease tissue density of microglia/macrophages and to inhibit microglial activation. This inhibition leads to a 54% reduction in microglial activation after 3 days if intra- or early post-ischemic hypothermic treatment in rats (subjected to a 2-hour MCAo) is maintained for 2 hours [153,162,168,179]. However, as the phagocytic activity of activated microglia and macrophages exerts beneficial effects in the long term, such parameters should be studied at later time points after the induction of hypothermia. Hypothermia also reduces BBB disruption occurring after stroke, which especially has implications for the passage of large molecules. Permeability to small molecules remains for at least 4 days post-injury [180]. Infiltration of neutrophils and monocytes can be reduced after transient MCAo with post-ischemic mild hypothermic treatment at 3 and 7 days post-injury [162]. This reduction can increase to approximately 75% if the hypothermic treatment is started 1-2 hours after a MCAo of 2 hours [157]. Similar results have been observed in other traumatic injury models [181].

Mild-to-moderate intra-ischemic hypothermia has a pan-inhibiting effect [23,182]. It reduces ATP depletion, anoxic depolarization, glutamate release, and apoptosis; maintains BBB integrity, inhibits white matter injury and blocks necrosis (if started during ischemia itself) [23,160,183]. The effects of hypothermia on inflammation show a more modulating response. However, hypothermia has been suggested to only delay neuronal damage rather than to provide permanent protection. Seven hours after a hypothermic treatment, a hyperthermic episode has been described which may revive or re-initiate pathophysiological mediators which had been quiet [159]. The challenge in hypothermic treatment is thus to know when to start cooling, how long to maintain it and how deep to cool. Deep cooling has no advantage over mild or moderate hypothermia, and actually causes more side effects [157]. Furthermore, a distinction has to be made between short and long cooling periods. It has been suggested that the disadvantages of delayed cooling could be overcome by performing a prolonged hypothermic protocol [22,182]. However, the modulating influence of hypothermia on neuroinflammation could also differ depending on these parameters and should be further investigated [184]. Depth, start of cooling and duration all influence outcome in both experimental and clinical settings, and make translation to the clinic difficult [185].

## Conclusions

The neuroinflammatory response after ischemic stroke involves several parameters, all connected with each other and connected to other pathways of the ischemic cascade. This makes it difficult to draw conclusions from research observations and to extrapolate them to clinical settings, because a limited number of differences between man and animal models may affect multiple levels of the inflammatory response. Many inflammatory components have neurotoxic as well as neuroprotective effects that may either stimulate or reduce cell damage after ischemic stroke. Inhibition of only one part of the neuroinflammatory response after ischemic stroke does not induce sufficient protection to improve recovery in patients. There is sufficient data that hypothermia acts at multiple levels of the ischemic cascade and of the neuroinflammatory response, and does not simply inhibit all processes induced by cerebral ischemia. It should be noted that hypothermia influences the expression of several inflammatory parameters at certain time points, but more research is required to discern which positive or negative effects contribute to neuroprotection. The ability of hypothermia to modulate many aspects of the inflammatory response may render translation to the clinic feasible. Another advantage of hypothermia could be the creation of a larger therapeutic time window to administer other neuroprotective agents and thus improve outcome after transient focal cerebral ischemia.

## Competing interests

The authors declare that they have no competing interests.

## Authors' contributions

The manuscript was written by AGC as part of her doctoral thesis. All authors provided editorial assistance and have read and approved the final version of the manuscript.
